# IBD Monitor: Romanian National Mobile Application for Inflammatory Bowel Disease Personalized Treatment and Monitoring

**DOI:** 10.3390/diagnostics12061345

**Published:** 2022-05-28

**Authors:** Carmen-Nicoleta Oancea, Răzvan-Cristian Statie, Dan-Ionuț Gheonea, Tudorel Ciurea, Mircea-Sebastian Șerbănescu, Costin-Teodor Streba

**Affiliations:** 1 Department of Analytical Chemistry, University of Medicine and Pharmacy of Craiova, 200349 Craiova, Romania; carmen.oancea@umfcv.ro; 2 Research Center of Gastroenterology and Hepatology, University of Medicine and Pharmacy of Craiova, 200638 Craiova, Romania; statierazvan@gmail.com (R.-C.S.); dan.gheonea@umfcv.ro (D.-I.G.); tudorel.ciurea@umfcv.ro (T.C.); 3 Department of Gastroenterology, University of Medicine and Pharmacy of Craiova, 200349 Craiova, Romania; costinstreba@gmail.com; 4 Department of Medical Informatics and Biostatistics, University of Medicine and Pharmacy of Craiova, 200349 Craiova, Romania; 5 Department of Pulmonology, University of Medicine and Pharmacy of Craiova, 200349 Craiova, Romania

**Keywords:** inflammatory bowel disease, patient support, mobile application, national registry

## Abstract

Background: In the last 30 years, we have seen an increase in the incidence of inflammatory bowel disease (IBD). Most cases are diagnosed in the 2nd and 3rd decades of life, a population group that is most familiar with the latest innovations in technology. Patients want to obtain more information about their disease and have complete control over the pathology, while reducing physical meetings with their doctor. Starting from these ideas, the present study aimed to develop a mobile application (app) to support IBD patients on symptoms/events reporting and on treatment administration monitoring. Methods: A multidisciplinary team was created to document and develop the app requirements and design its functionality. The app was beta-tested by several IBD patients. Their feedback was used to further refine the app. Results: We developed connected apps for both smartphones and smartwatches, with dedicated sections for event reporting and medication administration reminders/reporting. Conclusions: The development of apps dedicated to IBD patients is still in early progress. By creating this app, we aim to improve the evolution and compliance of IBD patients and to obtain new information that will have a beneficial impact on the management of these patients and open the door for personalized medicine.

## 1. Introduction

Inflammatory bowel disease (IBD) includes Crohn’s disease (CD) and ulcerative colitis (UC). These two pathologies of the digestive tract are characterized by a chronic evolution, marked by periods of remission and relapse, which require long-term monitoring, with frequent visits to the doctor in some patients. Therefore, the management of patients with CD or UC may sometimes be challenging. The continuous development of medical techniques has led to a faster and more reliable diagnosis of these conditions. In consequence, we have seen an increase in the incidence and prevalence of IBD, from 3.7 million patients in 1990, to over 6.8 million in 2017 [1]. Most cases are diagnosed in the 2nd and 3rd decades of life [2], a population group that is most familiar with the latest innovations in technology and has benefited most from the ”smartphone era”. In 2021, approximately 85% of the general population in medium-to-high-income countries owned a smartphone, of which those aged 18–29 in a percentage of 96%, and those aged 30–49 in a percentage of 95% [3].

As a result of facilitating internet access, in recent years we have faced more and more patients reading on the web about their symptoms and pathologies, of different therapeutic options and side effects, sometimes opting to modify or even halt the treatment on their own initiative, without asking their physician. This sometimes affects the results that physicians seek to achieve. Patients want to obtain more information concerning their illness, to somehow have complete control over the pathology, reducing as much as possible the physical meetings with their physician [4]. In addition, certain unfavorable situations, such as the global COVID-19 pandemic, have hampered chronic patients’ access to health care, and in many cases their monitoring has been inadequate. With the advancement of technology, we now have the option to put things in a doctor-friendly perspective, through telemedicine and the development of smartphone applications (apps) that meet the needs and desires of as many patients as possible.

Strategies such as treat-to-target (T2T) or IBD Prospect have been developed to meet the recommendation of the IBD Consensus on the Selection of Therapeutic Targets, according to which treatment goals have shifted from controlling only symptomatic diseases to support for compound therapeutic outcomes that can be periodically evaluated [5]. With such monitoring platforms, specialists in the field will come closer to therapeutic individualization for patients with IBD, but when the patient leaves the center, it remains for them to understand and follow exactly the treatment plan established by the doctor. Drug therapy is considered the cornerstone of obtaining remission and keeping symptoms under control. However, the chronic nature, relapses of the disease and the therapeutic relationship, as well as the clinical and psycho-social implications, can influence both the patient’s response and compliance with treatment. Thus, adherence to drug therapy is essential to maintain the condition in remission and to avoid possible consequences. Adherence has been defined as the way in which a person’s behavior (daily medication, lifestyle changes, regular investigations, medical examinations) corresponds to the indications of the attending physician. Therefore, various methods have been tried to increase adherence to IBD by reducing costs, simplifying techniques and improving cooperation between specialties, but also with the patient.

Medical data collecting and processing can greatly increase IBD treatment adherence, while refining strategies and driving management toward a deeply personalized experience. A large array of sensors exists, which can send ambient information (temperature, humidity, air pressure and light intensity etc.) to a smartphone/smartwatch-based solution, thus providing an overview of possible or emerging risk factors. In addition, most basic body measurements and biological data can be easily and securely collected through the sensors embedded in the mobile phone and smartwatch that the patient already uses—including heartrate, respiration rate, blood oxygen concentration, body temperature and even heart rhythm or blood pressure.

Artificial intelligence (AI) can be then used to analyze the data in a decentralized manner, conserving patient privacy while allowing critical decisions to be made in time, when abnormal readings are identified. The implementation of various types of data-privacy driven AI solutions that process sensitive data on site and only provide broad statistical indicators to physicians can nowadays be easily implemented into de-centralized decision-making systems that can direct a notification system for IBD patients.

Aim: The present study aimed to develop a mobile application (app) to support IBD patients on symptoms/events reporting and on treatment administration monitoring. In a data-wise manner, the app correctly identifies time-related symptoms, events, and treatment administration, making it suitable for data-mining algorithms on an individual/group base, and thus creating the context for personalized medicine, without omissions or distortions of events.

## 2. Methods

### 2.1. Research and Development Strategy

Having in mind the aim of the app, a mixed team was created, containing gastroenterologists, pathologists, pharmacists and data scientists. We have two active pediatricians involved in our IBD Management Center within our University who manage all issues related to this age group. The application developed within the project addresses patients above 16 years old; therefore, all aspects pertaining to the design of the app interface, types of collected data and implementation of active notifications were also validated by one of the two previously mentioned pediatricians. The gastroenterologists brought their experience on the disease and also their experience on the drawbacks of the original (face-to-face) approach. The pathologists suggested clinical data collections that could be of interest for the clinico-morphological data correlations. The pharmacists brought up their experience on auto-medication and treatment related symptoms. Last but not least, the data scientists integrated the team’s experience and added constraints on data acquisition and representation in order to make it suitable for data mining and thus creating the premises for personalized medicine, in compliance with the protocols for the treatment of inflammatory bowel diseases in Romania (http://cnas.ro/wp-content/uploads/2021/07/16.07.2021-lista-protocoalelor-terapeutice-IULIE-2021.pdf 30 April 2022).

The project was developed following the agile project management paradigm (https://www.atlassian.com/agile/project-management 10 February 2022), and Jira (https://www.atlassian.com/software/jira 10 February 2022) was used as an underlying software.

In the following chapters, we are going to introduce the activities and the technologies that made possible the development of our research.

### 2.2. Activities

The first activity of the project was documentation. First at an individual level, then within the team, everybody gathered information on IBD patients’ needs from a mobile app.

Next, the app requirements that resulted from the documentation of the medical part of the team were translated by the data scientists into epics and stories. Each epic was divided into tasks (stories). Each task was implemented, tested and refined.

Finally, the app was beta-tested by several patients and their feedback was used to further define the app.

### 2.3. Technologies

The IBD Monitor application is composed of two different pieces of software that interact with each other: the server-side central app and the mobile app.

The server app is used for storing the data and has two distinct backend interfaces. The first interface connects the national IBD register (https://www.registrul-ibd.ro/ 10 February 2022) and is the interface with the physician. Physicians can give access to their patients on the IBD Monitor app and can add treatments and monitor their patient’s reports directly from the IBD register. The second interface connects the mobile app, which gives the patient’s medication administration reminders and live report possibilities. The server-side implementation was conducted using PHP scripting language (https://www.php.net/ 10 February 2022) and having MariaDB Server as relational database management software. The server was hosted in a Romanian-based data center that offered high-performance and high-security hosting services.

The mobile app is used for medication administration reminders, treatment and event reporting. The app is fully patient-oriented and has the sole purpose of easing the patient’s way of reporting. The mobile app was developed in Android Studio Bumblebee 2021.1.1 (https://developer.android.com/studio/releases 10 February 2022) using the Windows version. The same software was used for the smartwatch app. The IBD Monitor app is publicly available via Google Play (https://play.google.com/store/apps/details?id=ro.umf.ibmmontior 5 May 2022), though it can only be used after the patients are enrolled by their physician.

## 3. Results

### 3.1. Details of the Project

As Romania is part of the EU, the General Data Protection Regulation (GDPR) (https://gdpr-info.eu/ 10 February 2022) applies to all the processed data. All patients obtain accurate information on how the data collected are used and are required to give a signed approval.

Since we are collecting medical information and this falls in the special category of personal data, further security measures were taken. First, the IBD Monitor app (both server and mobile side) uses completely anonymized data. For this, upon physician request, the IBD registry creates a unique session key that is passed to the IBD Monitor without any other information. This key identifies the patient within the IBD registry, but is blinded for the IBD Monitor app. All patients’ reports are carried out on this key. Moreover, the IBD Monitor and IBD register are located on separate servers, thus in case of a data leakage the information cannot be linked. The login itself is carried out by scanning a QR code containing the key, thus easing the user experience while securing the login.

### 3.2. App Description

The mobile app is constituted of two components: event reporting and medication administration reminders/reporting. An interactive menu can always be summoned by the patient on the left side of the screen. Menu sub-categories are Home, Reports, Treatment reports, Medication and Settings.

### 3.3. Home Screen

**Figure 1:**
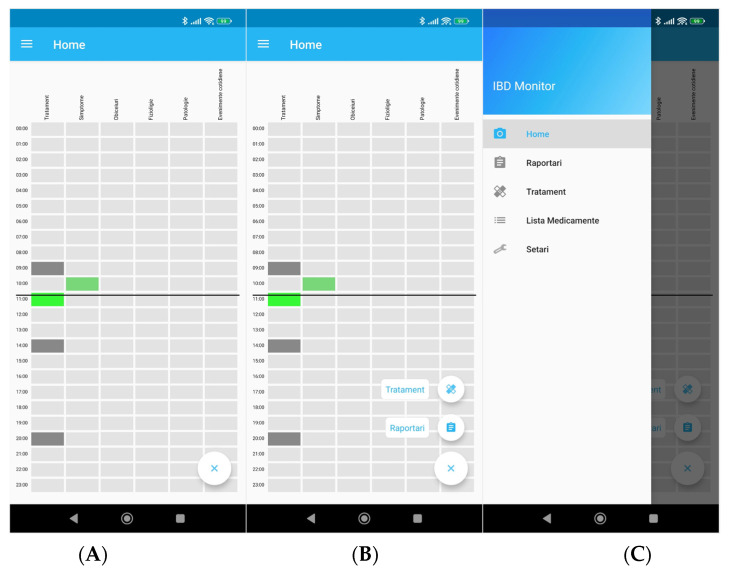
(**A**) Home screen, (**B**) home screen with add option, (**C**) main menu. Translation from top to bottom and from left to right. (**A**) Treatment, Symptoms, Customs, Physiology, Pathology, Daily events. (**B**) Treatment, Symptoms, Customs, Physiology, Pathology, Daily events, Treatment, Reports. (**C**) Home, Reports, Treatment, List of medicines, Settings.

The first screen the user sees when opening the app is the overall report visualization screen called the home screen (Figure 1A), where the patient has an overview of their reports and medication administration reminders. The section is organized as a timeline, divided in 24 lines that represent the hours of the day. A sliding ruler shows the user the current time of the day. The columns show individual report categories. The first column addresses the treatment, showing both medication reminders and reports, whereas the rest of the columns show only user reports on different reporting categories.

From the home screen, the user can add reports by pressing the add button (bottom right of Figure 1B), or by accessing the main menu of the app (Figure 1C).

### 3.4. Reporting Screen

**Figure 2:**
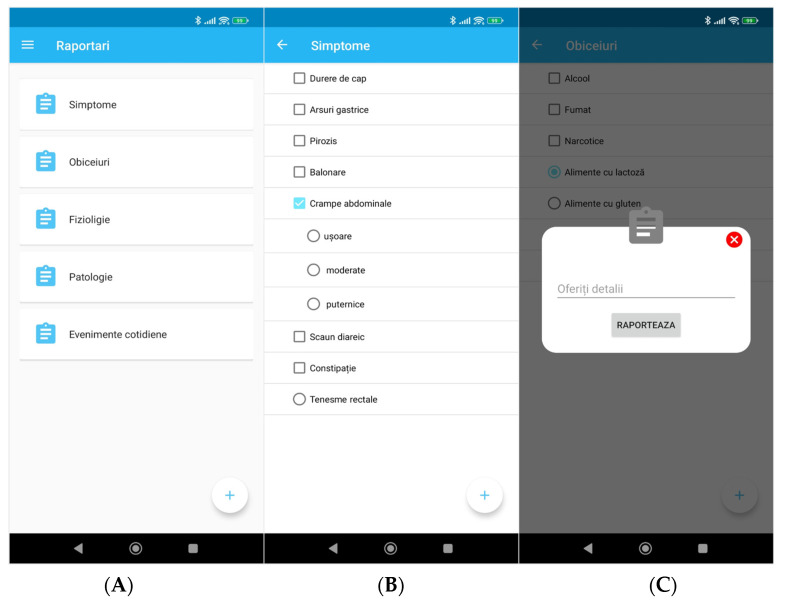
(**A**) Reports screen, (**B**) Symptoms screen, (**C**) Detailed report. (**A**) Symptoms, Customs, Physiology, Pathology, Daily events. (**B**) Symptoms, Headache, Heartburn, Bloating, Abdominal cramps (mild/moderate/severe), Diarrhea, Constipation, Rectal tenesmus. (**C**) Provide details, Report.

The reporting categories generated by the team are symptoms, habits, physiological, pathological and life events. All these categories were identified as relevant for IBD patients by the medical team and cannot be set freely by the patients, as it would bring inconsistency to the reporting. Each of the categories has two sub-levels that are also established by the medical team and the patients can add subjective comments detailing specifics of each report. For example, one reported symptom can be diarrhea (first sub-level of the Symptoms category), with one further sub-level, containing three options pertaining to the consistency of the stool: mucous, bloody or water diarrhea. Another example can be seen in Figure 2B.

No matter where the event reporting is triggered (add button or main menu), after accessing the command, the user will see all available reporting categories (Figure 2A). After selecting a specific category, a new view appears, and the user is prompted to select a report entity (Figure 2B). The report entities can have sub-entities as seen on the selection from Figure 2B or can be automatically reported, as the last element in Figure 2B. Different line icons identify the reports that have sub-reports options. Finally, for specific reports the user is prompted for details (Figure 2C). These are specifically identified details that are request by the physicians on a face-to-face meeting.

### 3.5. Medication Administration Reminders and Reporting

**Figure 3:**
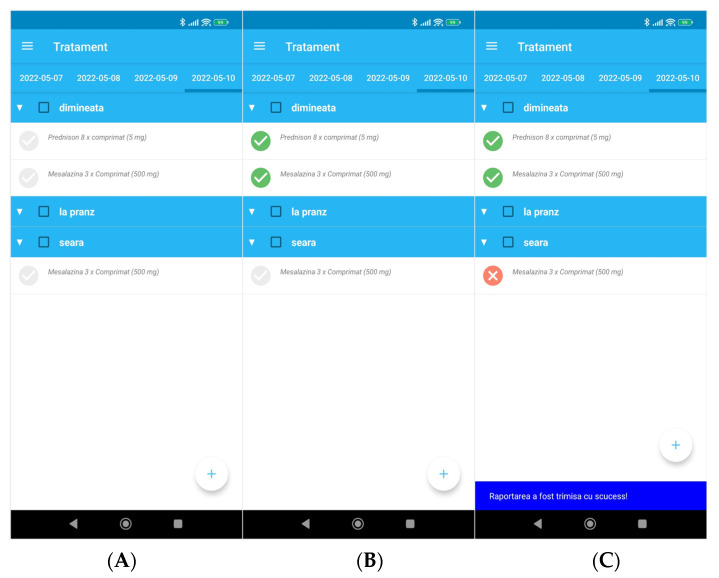
(**A**) Treatment screen, (**B**) confirmed administration of morning therapy, (**C**) unconfirmed medication of evening therapy. (**A**–**C**) Treatment: morning, noon, evening.

Medication reminders are sent to the patient as notifications. Notifications are sent up to three times a day, depending on the type of medication. The patient can define a preferred time for each notification. After acknowledging the notification, the patient is sent to the administration reporting view and can specify if they have taken the prescribed medication (Figure 3A). The patient can report on all medication at once with a single click or can choose to individually report on each prescribed product (Figure 3B). If the patient states that they did not take a specific product, they will have to give details on the reason through a dialog box similar to the one presented in Figure 2C. A seven-day history of medication reporting is available for the patient.

### 3.6. Other App Functions

**Figure 4:**
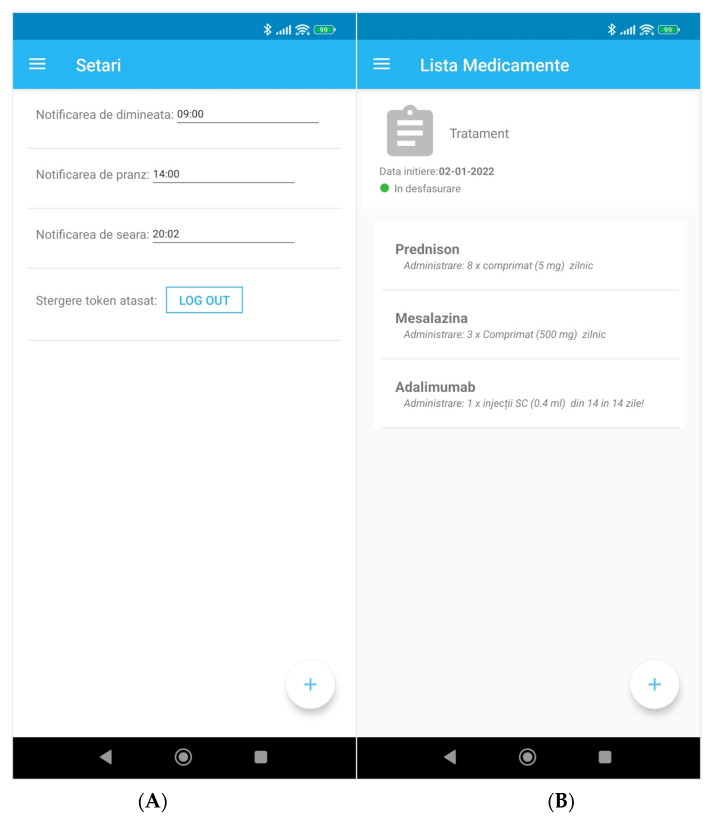
(**A**) Settings screen, (**B**) medication list. (**A**) Settings: times for morning notification, noon notification, evening notification, (**B**) list of medication, treatment, date of initiation, ongoing.

The app offers the possibility for the user to set a timing for the treatment administration notification (Figure 4A), thus increasing the app adoption and making it user-friendly.

Following the link in the main menu on the medication list, the patient has access to all of their prescribed products specifying all the administration details (product name, form, size and special precautions), can read and learn about them and can show them to the pharmacist if needed (Figure 4C). Because the user has no access to the medication (it can only be changed by the physician through the IBD register) this feature prevents auto-medication but encourages medical education.

### 3.7. The Smartwatch App

The mobile app is accompanied by a smartwatch app. The smartwatch app offers a reduced functionality of the mobile app and works concurrently. It was designed to be used whenever the mobile app use was considered inappropriate.

**Figure 5:**
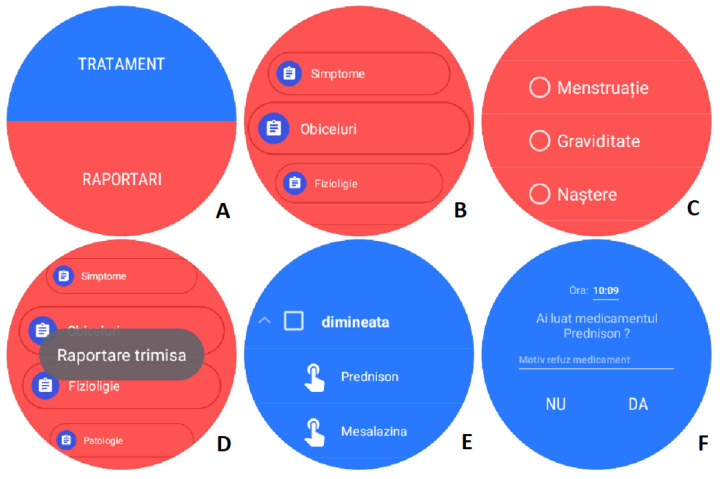
(**A**) Home screen, (**B**) Reports, (**C**) Physiology reports, (**D**) Confirmation of report, (**E**) Medication screen, (**D**) Details on unconfirmed administration. (**A**) Treatment, Reports. (**B**) Symptoms, Customs, Physiology. (**C**) Menstruation, Pregnancy, Birth. (**D**) Report sent. (**E**) Morning. (**F**) Did you take the medicine? Reason for refusing medicine, NO, YES.

Replicating the mobile app functionality, it offers two features: event reporting and medication administration reminders/reporting. Figure 5 shows detailed screens of the smartwatch app, main screen (Figure 5A), reports (Figure 5B) and report detail (Figure 5C), and treatment detail (Figure 5D).

## 4. Discussion

Telemedicine represents a viable alternative to the classic outpatient physical consultations, allowing efficient remote management of chronic patients. Thus, the advent of smartphones and smartwatches and the development of apps dedicated to patients with certain pathologies, create an appropriate framework for the implementation of this goal [6].

Studies show that IBD patients are interested in discovering new, up-to-date information about their pathology in order to control their disorder as effectively as possible [4]. Along these lines, especially young people and those with higher education levels are tempted to use the Internet to search for information about their pathology, even if the veracity of the information obtained is not always verified [7,8,9]. In addition, this group of patients prefers to obtain information through messaging platforms rather than from their doctor, at the expense of physical consultations [7,10].

A series of smartphone apps have been designed for patients with asthma or diabetes [11,12] to allow for monitoring of the evolution of the disease, as well as providing information for self-management of situations that require urgent resolution. The results obtained opened favorable prospects, but the development of a similar app for IBD patients is still in its infancy [6,7].

The benefits of using mobile devices in the care of patients with IBD may be represented by more efficient remote monitoring of patients, improved treatment compliance, adequate patient information about the disease and proper management, early warning of the physician in case of unfavorable evolution of the disorder and the eventual training of the patient regarding the self-management of some acute situations [7].

There are several studies that have evaluated smartphone apps dedicated to the management of patients with IBD available on the market at the time of the study [6,7]. A recent study dates from 2018 and evaluated 56 smartphone apps [7]. Features of the evaluated apps include symptom diaries (57%), medication administration reminders (36%), weight tracking (16%), nutrition diaries (36%), remote monitoring / surveying (14%), providing disease-related information (36%) and social media for IBD patients (9%). However, most apps do not have the ability to transmit real-time information to the patient’s attending physician. The study also concluded that most apps do not have clinical validation, and the involvement of competent medical staff in their development is low [7]. The app developed in our center was manufactured by a multidisciplinary team coordinated by a gastroenterologist.

Symptom diaries are a more effective alternative to paper-based diaries, facilitating clinical monitoring of patients by assessing severity scores, such as the Crohn’s disease activity index or the Harvey-Bradshaw index [6]. In order for patients to identify foods that may trigger the symptoms, diaries can be enhanced by the association of nutrition data [6,13].

Apps with a medication reminder function are proving useful in increasing treatment compliance, with studies showing that this function has a major impact in reducing cases of missed drugs [6,14,15,16,17]. Moreover, apps that provide up-to-date information about the disease can have favorable effects on the evolution of patients, as it has been shown that providing them with as much information about their illness as possible contributes to a greater interest from patients to strictly follow the recommendations and the treatment prescribed by their physician [7,18,19].

As for the apps that offer the function of social media for IBD patients, they can sometimes have beneficial effects on the patient, but they associate a big minus in certain situations, for instance, when patients with certain forms of IBD recommend treatments to those who have a different form of IBD, which can even have harmful consequences for the patient [4].

Although there are many smartphone apps for IBD patients, the number of studies evaluating their effectiveness in patient management is still low. A study conducted in Denmark on a group of 909 patients with IBD evaluated the effectiveness of an app called myIBDcoach [7,20]. A total of 465 patients were monitored through the app and 444 patients benefited from monitoring by classical methods. As follows, over a period of 12 months, in the group of patients who used telemedicine there was a reduction in outpatient visits to the doctor, as well as a reduction in the number of hospitalizations. However, the emergency visits, corticosteroid use and the need for surgeries were similar between the two groups [20].

The results of the clinical trial conducted by the Icahn School of Medicine at Mount Sinai, which evaluated the impact of the HealthPROMISE Mobile App on the evolution of 320 patients with IBD, are long awaited. The study assessed the time a patient spends using the app, the treatment changes initiated by physicians in response to patient feedback and the influence of the app on patients’ quality of life. The preliminary results obtained seem promising [7,21].

Some authors believe that patients’ adherence to treatment is improved when they become aware that the use of apps and the provision of data about their health condition in the app have a beneficial effect on the course of the malady. The more information doctors receive on patients’ status, the more they will be able to manage the disease more effectively. It is certain that the apps that will directly connect the patient and the attending physician will have a major impact on the management of patients with IBD [4].

### Current Limitations and Future Directions

Current limitations of the project pertain to the difficult implementation on different types of smartphones, as development in this ecosystem faces problems related to fragmentation and fundamentally different experiences for the user. However, later iterations of the embedded operating system will provide a more cohesive user experience, which will translate in a perfect integration of our apps.

A second limitation regards the smartwatch app, which currently runs only on Android-based smartwatch solutions. Different shapes of watch screens, multiple screen resolutions and various operating systems exist on this type of devices, making the user experience extremely non-cohesive. This will be resolved through extensive testing on a large cohort derived from the IBD Prospect register; this will also allow us to start implementing biomedical data from embedded sensors into the application.

The concrete application directions will consist of the macro implementation of the software solution developed through this research, by connecting it to IBD Prospect, with the possibility of obtaining relevant information regarding the epidemiology of inflammatory bowel disease, patient profile and knowledge of problems encountered by patients in adherence to prescribed treatments (conventional or biological/oral, subcutaneous, intravenous, topical). An iOS-compatible app is also in the works to cover all existing user groups. The unitarian implementation of this operating system will allow for a cohesive and uniform user experience, whereas the limited number of sensors available will be a possible impediment for future development of additional functions. However, this will be overcome by the use of external sensors that can be easily paired with the smartphone, providing data to our dedicated app.

## 5. Conclusions

The development of smartphone apps dedicated to IBD patients is still in early development and already-existing apps are quite far from meeting all the needs of patients. By creating this app, we aim to improve the evolution and compliance of IBD patients in our center and beyond, by maintaining an almost-permanent contact between patients and doctors, so that they enjoy a quality of life as close as possible of that of the general population. In addition, we aim to obtain new information that will have a beneficial impact on the management of IBD patients.

## Data Availability

The mobile app can be freely downloaded from https://play.google.com/store/apps/details?id=ro.umf.ibmmontior (5 May 2022) (in Romanian).
